# Reinfection rates, change in antibody titers and adverse events after COVID-19 vaccination among patients previously infected with COVID-19 in Metro Manila, Philippines: a secondary analysis of a completed cohort study

**DOI:** 10.1186/s12879-023-08743-6

**Published:** 2023-11-01

**Authors:** Carol Stephanie C. Tan-Lim, Ma. Liza Antoinette M. Gonzales, Leonila F. Dans, Cynthia P. Cordero, Marissa M. Alejandria, Eva C. Cutiongco dela Paz, Melissa A. Dator, Myzelle Anne J. Infantado-Alejandro, Maria Vanessa V. Sulit, Mary Ann D. Lansang

**Affiliations:** 1 Department of Clinical Epidemiology, College of Medicine, University of the Philippines Manila, Pedro Gil Street, Ermita, Manila Philippines; 2 Department of Pediatrics, College of Medicine, University of the Philippines Manila, Pedro Gil Street, Ermita, Manila Philippines; 3 Institute of Human Genetics, National Institutes of Health, University of the Philippines Manila, Pedro Gil Street, Ermita, Manila Philippines; 4 Institute of Clinical Epidemiology, National Institutes of Health, University of the Philippines Manila, Pedro Gil Street, Ermita, Manila Philippines

**Keywords:** COVID-19 vaccine, Antibody, Reinfection, Adverse events

## Abstract

**Background:**

Variation in immune response to COVID-19 vaccines is observed among different ethnicities. We aimed to describe the reinfection rates, change in antibody titers, and adverse events among Filipinos.

**Methods:**

This is a secondary analysis of a cohort study of 307 participants within one year of having COVID-19 infection. We measured COVID-19 antibody levels at pre-determined timepoints (Days 21, 90, 180, 270, and 360 from initial infection). We monitored for COVID-19 symptoms and obtained details on COVID-19 vaccination. An adjudication committee classified the participants as probable, possible, or unlikely COVID-19 reinfection. We determined the probable reinfection rate, adverse events, and the geometric mean titer (GMT) ratio of pre- and post-vaccination antibody levels according to type and brand of COVID-19 vaccine.

**Results:**

At the end of the follow-up period, 287 (93.5%) out of 307 study participants were fully vaccinated, 1 was partially vaccinated (0.3%), and 19 were unvaccinated (6.2%). Among the fully vaccinated participants, those given mRNA vaccines had the lowest reinfection rate (19.2 cases/100 person-years, 95% CI 9.6, 38.4), followed by viral vector vaccines (29.8 cases/100 person-years, 95% CI 16.9, 52.4). We observed the highest reinfection rate among those given inactivated virus vaccines (32.7 cases/100 person-years, 95% CI 23.6, 45.3). The reinfection rate was 8.6 cases/100 person-years (95% CI 4.1, 17.9) for unvaccinated participants and 3.6 cases/100 person-years (95% CI 0.5, 25.3) for partially vaccinated participants. We observed the largest rise in antibody titers among those given mRNA vaccines (GMT ratio 288.5), and the smallest rise among those given inactivated virus vaccines (GMT ratio 16.7). We observed the highest percentage of adverse events following immunization with viral vector vaccines (63.8%), followed by mRNA vaccines (62.7%), and the lowest for inactivated virus vaccines (34.7%). No serious adverse events were reported.

**Conclusion:**

Vaccinees given the mRNA vaccines had the lowest reinfection rate and the highest rise in antibody titers. Vaccinees given inactivated virus vaccines had the highest reinfection rate, smallest rise in antibody titers, and lowest percentage of adverse events. The small sample size and imbalanced distribution of the type of vaccines received limits the external generalizability of our results.

**Study Registration:**

The cohort study was registered at the Philippine Health Research Registry on December 14, 2020 (PHRR201214-003199).

**Supplementary Information:**

The online version contains supplementary material available at 10.1186/s12879-023-08743-6.

## Introduction

The development of coronavirus disease 2019 (COVID-19) vaccines greatly altered the course of the pandemic. Vaccines prevented 14.4 million deaths (95% credible interval 13.7 to 15.9 million) globally in the first year of vaccine administration [1].

There are several types of COVID-19 vaccines, such as viral vector vaccines, nucleic acid vaccines, protein subunit vaccines, and inactivated whole virus vaccines. There are advantages and disadvantages for each vaccine type related to its immunogenicity, production, and stability [2].

The national COVID-19 vaccination program of the Philippines began in March 2021. The vaccines that received emergency use authorization approval in the Philippines include mRNA vaccines BNT162b2 (by Pfizer/BioNTech) and mRNA-1273 (by Moderna); non-replicating viral vectors AZD1222 (by Oxford/AstraZeneca), Sputnik V (by Gamaleya), and Ad26.COV2.S (by Janssen); and inactivated viruses CoronaVac (by Sinovac), inactivated Vero Cells (by Sinopharm), and Covaxin (by Bharat Biotech). These vaccines are given as a 2-dose primary series, except Ad26.COV2.S, which is given as a single dose primary series [3]. As of March 2023, only monovalent vaccines are available in the Philippines.

Several studies evaluated the effectiveness and safety of the different COVID-19 vaccines. A 2023 systematic review showed high vaccine effectiveness (VE) of primary series of any COVID-19 vaccine at 14–42 days from vaccination (VE 92%, 95% confidence interval [CI] 88, 94% for hospitalization; VE 91% (95% CI 85, 95%) for mortality). Analysis by type of vaccine showed that VE against COVID-19 infection was higher for mRNA vaccines (VE 87%, 95% CI 84, 90%) compared to viral vectors vaccines (VE 69%, 95% CI 60, 75%). Analysis by brand showed that highest VE for mRNA-1273 (VE 92%, 95% CI 88, 94%), followed by BNT162b2 (VE 86%, 95% CI 81, 89%), AZD1222 (VE 72%, 95% CI 61, 79%) and Ad26.COV2.S (VE 61%, 95% CI 48, 70%) [4].

A network meta-analysis published in 2022 assessed the effectiveness in preventing COVID-19 infection and safety of 28 vaccines. The lowest relative risk (RR) for infection was observed for BNT162b2 (RR 0.05, 95% CI 0.03, 0.10; compared to placebo). The most common local side effect reported was pain, while the most common systemic side effects were fever and fatigue. Sinopharm and V-01 vaccines were found to be the safest in terms of local and systemic side effects [5].

Another network meta-analysis published in 2022 demonstrated that mRNA-1273 resulted in the largest increase in neutralizing antibodies levels (SMD 1,605.34, 95% CI 1,534.68, 1,676.00). Only mRNA-1273 had significantly increased risk of systemic adverse reactions compared to placebo (RR 6.69, 95% CI 3.82, 11.71) [6].

Age, comorbidities and ethnicity result in significant variations in immune response to COVID-19 vaccines [7]. There are no published studies that compare the effectiveness, immunogenicity, and safety of the different types of vaccines in the Filipino population. In 2021 to 2022, we completed a cohort study to monitor the antibody levels of Filipino patients in Metro Manila, Philippines who were diagnosed with COVID-19. We observed if they were reinfected within one year from the initial infection. Study participants received different types of COVID-19 vaccine during the study period [8]. Using data obtained from this completed cohort study, we aimed to describe the reinfection rates, change in antibody titers, and adverse events in a cohort of Filipino adults previously infected with COVID-19 infection who received different types of COVID-19 vaccines. Findings of this study may be used to guide policies on vaccine recommendations and address vaccine hesitancy in the Philippines.

## Methods

### Study Design

This study is a secondary analysis of COVID-19 vaccination data gathered in a previously completed cohort study [8]. The full methodology of the completed cohort study is in Appendix 1. We conducted the cohort study from March 2021 to July 2022 to determine the durability of antibodies among 307 study participants with COVID-19 infection in Metro Manila, Philippines. We measured antibody levels on days 21, 90, 180, 270, and 360 from onset of symptoms, or positive reverse transcription-polymerase chain reaction (RT-PCR) test for participants with asymptomatic infection. We used a laboratory-based semi-quantitative electrochemiluminescence immunoassay (ECLIA) test (Elecsys® Anti-SARS-CoV-2 S assay) to measure antibody levels. This test detected the RBD-specific total antibody levels (IgG, IgA, IgM). The lower limit of detection of this test is 0.4 U/mL, while the upper limit of detection is 250 U/mL. For study participants who had results < 0.4 U/mL, we recorded the result as 0.39 U/mL to facilitate mathematical computation and data analysis. For study participants with results > 250 U/mL, we performed serial dilutions as necessary to increase the upper limit of detection to up to 250,000 U/mL.

We monitored the cohort through phone calls every two weeks for one year to inquire if they developed symptoms consistent with COVID-19, including fever, cough, difficulty of breathing, fatigue, muscle or body aches, headache, loss of taste, loss of smell, sore throat, nasal congestion, rhinorrhea, diarrhea, nausea and vomiting. We also asked participants if they received the COVID-19 vaccine, and the type, brand and date of vaccination.

### Study Population

This study used the same inclusion and exclusion criteria of the cohort study as follows:

#### Inclusion criteria

(1) Adult (≥ 18 years old); (2) Diagnosed with COVID-19 through RT-PCR, including patients with asymptomatic, mild, moderate, severe or critical disease; (3) Within 21 days since onset of symptoms (if symptomatic) or since RT-PCR positivity (if asymptomatic); (4) Owned a mobile phone; (5) Permanent address within Metro Manila; and (6) Able to provide informed consent.

#### Exclusion criteria

(1) Received COVID-19 vaccine prior to enrolment in the cohort study; and (2) received or intended to receive convalescent plasma or intravenous immunoglobulin during the follow-up and monitoring period .

### Study procedures

#### Description of the reinfection rates and adverse events of different types of COVID-19 vaccine

In the cohort study [8], an adjudication committee classified study participants as probable, possible or unlikely to have COVID-19 reinfection based on demographic information, relevant medical history, antibody levels before and after symptoms occurred, RT-PCR test results and cycle threshold values (if available) and vaccination status. The criteria for probable reinfection consisted of (1) clinically compatible symptoms (any of the following: fever or chills, cough, difficulty breathing or shortness of breath, fatigue, muscle or body aches, headache, sore throat, new loss of taste or smell, congestion or runny nose, nausea or vomiting, diarrhea), and (2) positive RT-PCR done at least 3 months after recovery, or positive antigen test, or increase in antibody levels not otherwise explained by vaccination. Confirmation by genomic testing could not be in the cohort study.

This study described the rates of probable reinfection among study participants according to type and brand of COVID-19 vaccine received. We also described reinfection rates among unvaccinated and partially vaccinated study participants as a point of comparison. We extracted data from all study participants.

We described adverse events following immunization, defined as any untoward medical event occurring up to 14 days after vaccination but not necessarily having a causal relationship to the vaccine, for each type and brand of vaccine. These were further classified into local, systemic, and serious adverse events [9]. We extracted data from all study participants who received at least 1 dose of COVID-19 vaccine.

#### Description of the change in antibody titers of the different types and brands of COVID-19 vaccine

We described the change in antibody titers by obtaining the ratio of the post- and pre-vaccination titers of the participants. The pre-vaccination titer refers to the antibody titer extracted prior to vaccination, while the post-vaccination titer refers to the antibody titer extracted at least 2 weeks after vaccination. We also reported the time interval from antibody titer determination to vaccination.

In the analysis for the primary vaccine series, we extracted data from all individuals who were fully vaccinated, defined as receipt of at least the primary vaccine series. The primary series is a 2-dose series of the same COVID-19 vaccine brand (except for Ad26.COV2.S which is given as a single dose). For individuals who subsequently received booster doses, we used the antibody determination done at least 2 weeks after the primary series vaccination but before the administration of the booster dose as the post-vaccination titer. We compared the post-and pre-vaccination titers according to type and brand of primary vaccine series.

In the analysis of the booster regimens, we extracted data from all individuals who received at least 1 booster dose. For individuals who received 2 booster doses, we used the antibody level extracted at least 2 weeks after the first booster dose to allow comparisons with the majority of the participants who received just 1 booster dose. We compared the post- and pre-vaccination titers according to the type of primary vaccine series and whether the booster vaccine was homologous or heterologous. We defined heterologous booster regimens as receipt of a different vaccine brand for booster compared to the primary series.

### Data Analysis

We extracted pertinent data from the password-secured files of the completed cohort study. We used MS Excel for data management and STATA 14.0 for data analysis.

We reported antibody titers as geometric mean titers (GMT) with geometric standard deviation (GSD). We determined the GMT ratio, computed as the post-vaccination titer divided by the pre-vaccination titer, for each type and brand of COVID-19 vaccine administered.

## Results

### Sociodemographic profile of study participants

The cohort consisted of 164 females (53.4%) and 143 males (46.6%), with a median age of 36 years (interquartile range [IQR] 19). The severity of their initial COVID-19 infection was mild for 170 participants (55.4%), moderate for 24 participants (7.8%), severe for 28 participants (9.1%), critical for 7 participants (2.3%), and asymptomatic for 78 participants (25.4%).

After one year of follow-up, 287 (93.5%) were fully vaccinated, 1 was partially vaccinated with CoronaVac (0.3%), and 19 were unvaccinated (6.2%). There were 220 participants (71.7%) who received booster doses, of which 217 participants received one booster dose, and 3 participants received two booster doses.

Among the 287 fully vaccinated participants, the most common type of primary series vaccine received was inactivated virus vaccine (54.7%, n = 157). There were 66 participants (23.0%) who received non-replicating viral vector vaccines, and 64 (22.3%) who received mRNA vaccines. The most common brand received was CoronaVac by Sinovac (54.4%, n = 156), followed by AZD1222 by Oxford/ AstraZeneca (16.0%, n = 46), and BNT162b2 by Pfizer/BioNTech (13.9%, n = 40).

Among those who received a booster, 168 (76.4%) received mRNA vaccine, 39 (17.7%) received non-replicating viral vector vaccine, and 13 (5.9%) received inactivated virus vaccines. Table 1 shows the detailed frequency distribution of study participants by brand of vaccine received.

**Table 1:** Brands of COVID-19 vaccine received by study participants (n = 287)

Brand of vaccine received as primary series	Frequency (%)	Booster	Frequency
Inactivated viruses			
CoronaVac (Sinovac)	156 (54.4)	CoronaVac	13
		AZD1222	23
		BNT162b2	38
		mRNA-1273	49*
Inactivated Vero Cells (Sinopharm)	1 (0.3)	None	0
Non-replicating viral vectors			
AZD1222 (Oxford/ AstraZeneca)	46 (16.0)	AZD1222	9
		BNT162b2	15
		mRNA-1273	11
Sputnik V (Gamaleya)	11 (3.8)	AZD1222	1
		BNT162b2	6
		mRNA-1273	3
Ad26.COV2.S (Janssen)	9 (3.1)	AZD1222	1
		BNT162b2	2
		mRNA-1273	1
mRNA vaccines			
BNT162b2 (Pfizer/BioNTech)	40 (13.9)	AZD1222	2
		BNT162b2	22
		mRNA-1273	9
mRNA-1273 (Moderna)	24 (8.4)	AZD1222	3
		BNT162b2	6
		mRNA-1273	6
Total	**287**		**220**

*3 participants received 2 booster doses

The rate of probable reinfection among unvaccinated participants was 8.6 cases per 100 person-years (95% CI 4.1, 17.9), while the rate among partially vaccinated participants was 3.6 cases per 100 person-years (95% CI 0.5, 25.3). Among the fully vaccinated participants, we observed the lowest rate of reinfection among those given mRNA vaccines as primary series at 19.2 cases per 100 person-years (95% CI 9.6, 38.4). Participants who received inactivated virus vaccines had the highest reinfection rate at 32.7 cases per 100 person-years (95% CI 23.6, 45.3) (Table 2).

By vaccine brand, we observed the highest reinfection rate among participants who received Sputnik V at 63.2 cases per 100 person-years (95% CI 26.3, 151.7), followed by CoronaVac at 32.9 cases per 100 person-years (95% CI 23.7, 45.5). Participants who received BNT162b2 had a reinfection rate of 14.2 cases per 100 person-years (95% CI 5.3, 37.9). There was no identified case of probable reinfection among those given Ad26.COV2.S and inactivated Vero cell vaccines.

**Table 2:** Reinfection rates among participants according to type and brand of COVID-19 vaccine received

Primary series vaccine	Person-years of observation	Number of cases with probable reinfection	Rate per 100 person-years	95% Confidence Interval
Inactivated viruses	110.1	36*	32.7	23.6, 45.3
CoronaVac (Sinovac)	109.5	36*	32.9	23.7, 45.5
Inactivated Vero Cells (Sinopharm)	0.5	0	0	
Non-replicating viral vectors	40.3	12**	29.8	16.7, 52.4
AZD1222 (Oxford/ AstraZeneca)	27.0	7	25.9	12.3, 54.3
Sputnik V (Gamaleya)	7.9	5**	63.2	26.3, 151.7
Ad26.COV2.S (Janssen)	5.3	0	0	
mRNA vaccines	41.7	8	19.2	9.6, 38.4
BNT162b2 (Pfizer/BioNTech)	28.2	4	14.2	5.3, 37.9
mRNA-1273 (Moderna)	13.5	4	29.6	11.1, 78.9
Partially vaccinated	28.1	1	3.6	0.5, 25.3
Unvaccinated	81.8	7	8.6	4.1, 17.9

*4 participants received 1 booster dose (mRNA vaccine) at the time of reinfection

**1 participant received 1 booster dose (mRNA vaccine) at the time of reinfection

### Antibody titers

Table 3 shows the GMT ratios of post- and pre-vaccination RBD-specific antibody titers according to type and brand of COVID-19 vaccine received. We observed the largest rise in antibody titer after primary series vaccination with mRNA vaccines (GMT ratio 288.5), followed by non-replicating viral vector vaccines (GMT ratio 97.2). We observed the smallest rise in antibody levels after primary series vaccination with inactivated virus vaccines (GMT ratio 16.7). The average interval from antibody titer determination to vaccination was similar across the 3 types of vaccines, ranging from 31.0 to 35.0 days (SD 18.1 to 24.5) from the pre-vaccination titer determination to administration of the first vaccine dose, and 54.6 to 63.0 days (SD 21.7 to 25.5) from the administration of the second vaccine dose (first dose for participants who received Ad26.COV2.S) to post-vaccination titer determination.

By vaccine brand, we observed the largest rise in antibody titer after primary series vaccination with BNT162b2 (GMT ratio 302.8), followed by mRNA-1273 (GMT ratio 265.5), and Sputnik V (GMT ratio 232.3). We observed the smallest rise in antibody level after primary series vaccination with inactivated Vero Cells (GMT ratio 2.4, n = 1). We observed the second lowest rise in antibody levels after primary series vaccination with CoronaVac (GMT ratio 16.9).

The average interval from pre-vaccination titer determination to vaccination varied across the vaccine brands, ranging from 26.0 days (AZD1222) to 67 days (inactivated Vero Cells). The average time interval from administration of the second vaccine dose (first dose for participants who received Ad26.COV2.S) to post-vaccination titer determination ranged from 46.0 days (Ad26.COV2.S) to 93 days (inactivated Vero Cells).

**Table 3:** Total RBD-specific immunoglobulin levels of study participants according to type and brand of COVID-19 vaccine received

Vaccine Received as primary series	Pre-vaccination titer (U/mL) GMT (GSD)	Post-vaccination titer (U/mL) GMT (GSD)	GMT ratio	Interval in days from pre-vaccina- tion titer to vaccination Mean (SD)	Interval in days from vaccination to post-vaccina- tion titer Mean (SD)
Inactivated viruses (n = 157)	31.2 (9.3)	520.1 (3.8)	16.7	35.0 (21.1)	59.8 (23.4)
CoronaVac (Sinovac) (n = 156)	31.0 (9.4)	522.4 (3.8)	16.9	34.0 (21.0)	60.0 (23.4)
Inactivated Vero Cells (Sinopharm) (n = 1)	106.9 (N/A)	261.4 (N/A)	2.4	67 (N/A)	93 (N/A)
Non-replicating viral vectors (n = 66)	22.6 (10.8)	2,196.0 (2.7)	97.2	31.0 (18.1)	54.6 (21.7)
AZD1222 (Oxford/ AstraZeneca) (n = 46)	18.6 (9.8)	1,854.3 (2.4)	99.7	26.0 (16.8)	53.0 (20.3)
Sputnik V (Gamaleya) (n = 11)	9.3 (7.5)	2,160.4 (2.4)	232.3	42.0 (17.2)	67.0 (25.6)
Ad26.COV2.S (Janssen) (n = 9)	180.5 (9.9)	5,174.4 (3.3)	28.7	38.0 (18.8)	46.0 (19.7)
mRNA vaccines (n = 64)	23.3 (9.1)	6,722.9 (3.1)	288.5	35.0 (24.5)	63.0 (25.5)
BNT162b2 (Pfizer/BioNTech) (n = 40)	16.9 (9.1)	5,117.2 (3.1)	302.8	35.0 (26.3)	63.0 (26.7)
mRNA-1273 (Moderna) (n = 24)	40.7 (8.6)	10,806.8 (2.6)	265.5	36.0 (21.5)	61.8 (24.0)

**Fig. 1:**
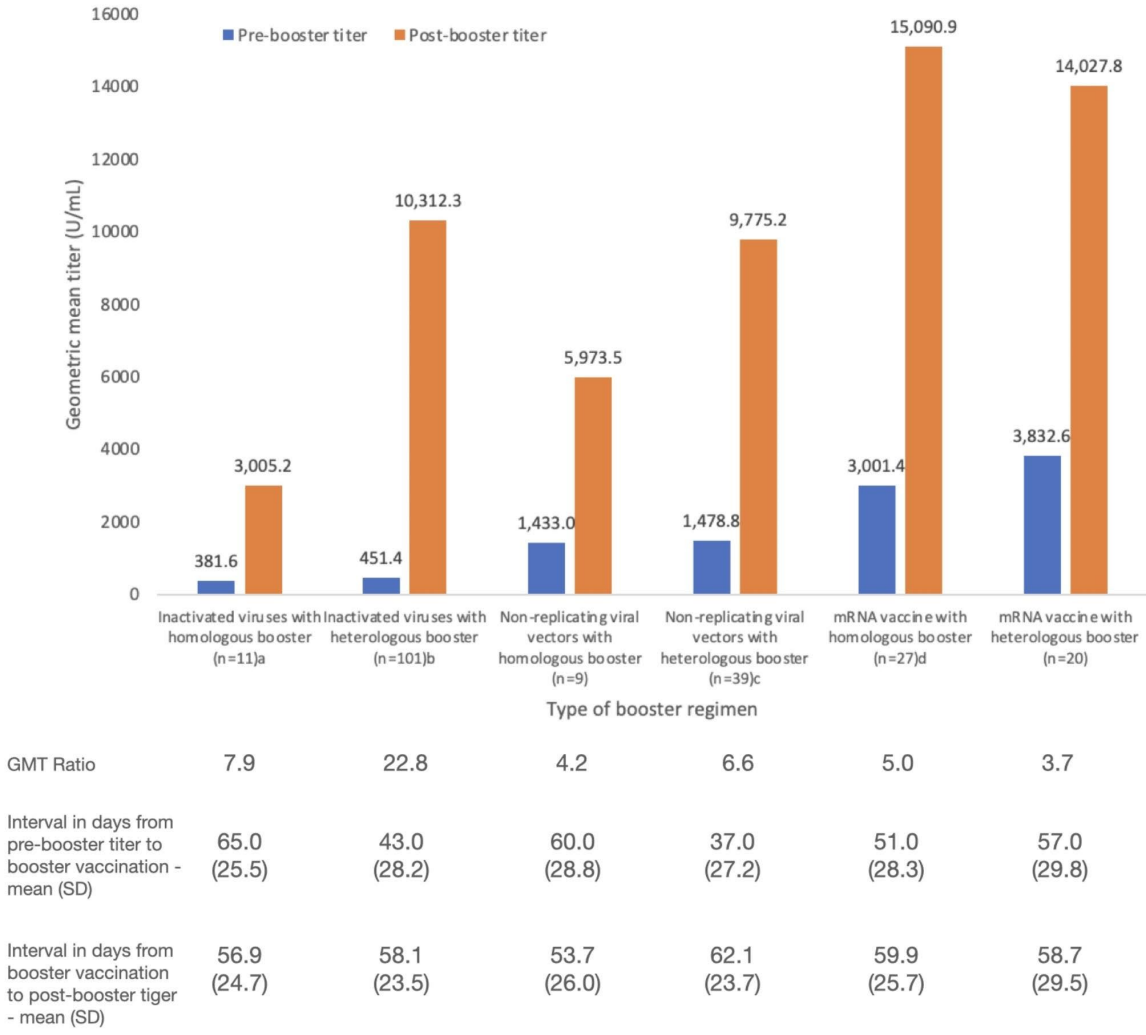
Total RBD-specific immunoglobulin levels of study participants according to booster regimen received. ^a^ 2 participants without a post-booster titer. ^b^ 9 participants without a post-booster titer. ^c,d^ 1 participant without a post-booster titer

The GMT ratios of post- and pre-booster RBD-specific antibody titers according to the type of booster regimen is shown in Fig. 1 (n = 207). We observed the largest increase in antibody titers among participants given inactivated virus vaccine as primary series: GMT ratio of 22.8 for inactivated viruses with heterologous booster (mRNA or viral vector vaccine) and GMT ratio 7.9 for those with homologous booster. Among those given non-replicating viral vectors as primary series, the rise in antibody titers was greater for heterologous boosters (GMT ratio 6.6) compared to homologous boosters (GMT ratio 4.2). Among those given mRNA vaccines as primary series, the rise in antibody titers was greater for homologous boosters (5.0) compared to heterologous boosters (3.7).

### Adverse events

Table 4 shows the adverse events following immunization according to type and brand of COVID-19 vaccine received. We observed the highest percentage of adverse events following immunization with non-replicating viral vector vaccines (63.8%), closely followed by mRNA vaccines (62.7%). Only 34.7% of study participants who received inactivated virus vaccines experienced adverse events.

By vaccine brand, we observed the highest percentage of adverse events following immunization with mRNA-1273 (74.6%), followed by Sputnik V (63.6%) and AZD1222 (60.3%). No adverse events were reported among the 2 participants who received inactivated Vero cells vaccine, while only 34.9% of those given CoronaVac reported adverse events.

**Table 4:** Adverse events of the different types of COVID-19 vaccines (n = 288 participants)

Type and brand of vaccine	Total adverse events reported n/N^a^ (%)	Adverse events reported after first dose n/N (%)	Adverse events reported after second dose n/N (%)	Adverse events reported after booster dose n/N (%)
Inactivated viruses	113/326 (34.7)	59/157 (37.6)	52/157 (32.5)	2/12 (16.7)
CoronaVac (Sinovac)	113/324 (34.9)	59/156 (37.6)	52/156 (33.3)	2/12 (16.7)
Inactivated Vero Cells (Sinopharm)	0/2 (0)	0/1 (0)	0/1 (0)	N/A
Non-replicating viral vectors	97/162 (63.8)	48/66 (72.7)	26/57 (47.4)	23/39 (59.0)
AZD1222 (Oxford/ AstraZeneca)	79/131 (60.3)	35/46 (76.1)	21/46 (45.6)	23/39 (59.0)
Sputnik V (Gamaleya)	14/22 (63.6)	9/11 (81.8)	5/11 (45.5)	N/A
Ad26.COV2.S (Janssen)	4/9 (44.4)	4/9 (44.4)	N/A	N/A
mRNA vaccines	185/295 (62.7)	39/65 (60.0)	38/65 (58.4)	108/165^b^ (65.4)
BNT162b2 (Pfizer/BioNTech)	88/165 (53.3)	20/40 (50.0)	24/40 (60.0)	44/85 (51.8)
mRNA-1273 (Moderna)	97/130 (74.6)	19/25 (76.0)	14/25 (56.0)	64/80^b^ (80.0)

^a^ denominator is total number of doses administered

^b^ 3 doses given as fourth dose

N/A – no dose administered

There were no serious adverse events reported by the study participants. We observed the highest percentage of systemic reactions among those given mRNA vaccines. Of the 295 doses of mRNA vaccines administered, 121 (41.0%) resulted in systemic reactions. Of the 162 doses of non-replicating viral vector vaccines administered, 65 (40.1%) resulted in systemic reactions. We observed the lowest percentage of systemic reactions among participants given inactivated virus vaccines. Of the 326 doses of inactivated virus vaccines administered, only 46 (14.1%) resulted in systemic reactions.

The most common systemic reaction was fever for non-replicating viral vectors (27.8%) and mRNA vaccines (18.6%). The most common systemic reactions were drowsiness and headache for inactivated virus vaccines (4.9%). For all vaccine types, local reactions included pain on injection site and arm heaviness. The specific types of systemic and local reactions are summarized in Table 5. Several participants reported more than 1 type of reaction.

**Table 5:** Types of adverse events of the different types of COVID-19 vaccines (participants may report more than 1 reaction; n = 288 participants)

Adverse events	Type of Vaccine		
	Inactivated viruses	Non-replicating viral vectors	mRNA vaccines
	N = 326 doses	N = 162 doses	N = 295 doses
	**n (%)**	**n (%)**	**n (%)**
Systemic Reactions			
Drowsiness	16 (4.9)	1 (0.6)	0
Headache	16 (4.9)	18 (11.1)	30 (10.2)
Body pain/myalgia	8 (2.5)	26 (16.0)	50 (16.9)
Fatigue	8 (2.5)	13 (8.0)	30 (10.2)
Fever	3 (0.9)	45 (27.8)	55 (18.6)
Diarrhea	1 (0.3)	0	1 (0.3)
Chills	1 (0.3)	7 (4.3)	8 (2.7)
Nausea	0	0	3 (1.0)
Local Reactions			
Pain at injection Site	56 (17.2)	36 (22.2)	71 (24.1)
Arm heaviness	20 (6.1)	3 (1.9)	16 (5.4)

*Denominator is total number of doses administered to participants

Considering the individual vaccine brands, we observed the highest percentage of systemic reactions among those who received mRNA-1273. Of the 130 doses of mRNA-1273 vaccines administered, 76 (58.5%) were associated with systemic reactions. This was followed by AZD1222 (55/131, 42.0%); Sputnik V (8/22, 36.4%); BNT162b2 (45/165, 27.2%); and Ad26.COV2.S (2/9, 22.2%). We observed the lowest percentage of systemic reactions among those given CoronaVac, with 46 out of the 324 doses administered resulting in systemic reactions (14.2%).

The most common systemic reaction was fever for mRNA-1273 (30.8%), AZD1222 (30.5%) and Sputnik V (18.2%). Body pain or myalgia was the most frequent systemic reaction for BNT162b2 (11.5%), while drowsiness and headache were the most frequent systemic reactions for CoronaVac (4.9%). Local reactions included pain on injection site and arm heaviness for CoronaVac, AZD1222, BNT162b2, and mRNA-1273, and pain on injection site for Sputnik V and Ad26.COV2.S. The specific types of systemic and local reactions are summarized in Table 6. Several participants reported more than 1 type of reaction.

**Table 6:** Types of adverse events of the individual brands of COVID-19 vaccines (participants may report more than 1 reaction; n = 288 participants)

Adverse events	Brand of Vaccine					
	CoronaVac (Sinovac)	AZD1222 (Oxford/ AstraZeneca)	Sputnik V (Gamaleya)	Ad26.COV2.S (Janssen)	BNT162b2 (Pfizer/BioNTech)	mRNA-1273 (Moderna)
	N = 324 doses	N = 131 doses	N = 22 doses	N = 9 doses	N = 165 doses	N = 130 doses
	**n (%)**	**n (%)**	**n (%)**	**n (%)**	**n (%)**	**n (%)**
Systemic Reactions						
Drowsiness	16 (4.9)	0	1 (4.5)	0	0	1 (0.8)
Headache	16 (4.9)	14 (10.7)	4 (18.2)	0	11 (6.7)	19 (14.6)
Body pain/myalgia	8 (2.5)	24 (18.3)	1 (4.5)	1 (11.1)	19 (11.5)	31 (23.8)
Fatigue	8 (2.5)	11 (8.4)	1 (4.5)	1 (11.1)	8 (4.8)	22 (16.9)
Fever	3 (0.9)	40 (30.5)	4 (18.2)	1 (11.1)	15 (9.1)	40 (30.8)
Diarrhea	1 (0.3)	0	0	0	1 (0.6)	0
Chills	1 (0.3)	6 (4.6)	1 (4.5)	0	3 (1.8)	5 (3.8)
Nausea	0	0	0	0	1 (0.6)	2 (1.5)
Local Reactions						
Pain at injection Site	56 (17.3)	25 (19.1)	9 (40.9)	2 (22.2)	41 (25.9)	30 (23.1)
Arm heaviness	20 (6.2)	3 (2.3)	0	0	8 (4.9)	8 (6.2)

*Denominator is total number of doses administered to participants

## Discussion

We found that study participants who received mRNA vaccines as primary series had the lowest reinfection rate and the highest increase in antibody titers. Those who received inactivated virus vaccines had the highest reinfection rate and the lowest rise in antibody titers. In terms of individual brands of COVID-19 vaccine, there were no identified cases of probable reinfection among participants given inactivated Vero Cells and Ad26.COV2.S vaccines as primary series. However, these two brands had the lowest number of recipients (only one and nine participants received the inactivated Vero cells vaccine and Ad26.COV2.S vaccines, respectively). We observed the highest reinfection rate among vaccinees who received Sputnik V, followed by CoronaVac.

We noted that the highest reinfection rate was observed among participants who received Sputnik V (Gamaleya) despite the large rise in antibody titer after primary series vaccination. This may be due to other variables that influence reinfection rates, including age, co-morbidities, employment, and exposure to COVID-19 [10].

**Fig. 2:**
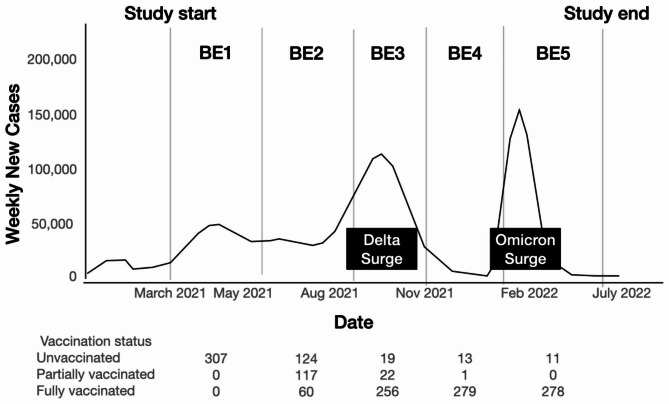
Epidemiological context in the Philippines and the vaccination status of study participants. (Image modified from the https://doh.gov.ph/covid19tracker) [11]. BE1 = first blood extraction at day 21, BE2 = second blood extraction at day 90, BE3 = third blood extraction at day 180, BE4 = fourth blood extraction at day 270, BE5 = fifth blood extraction at day 360,

The reinfection rates of the unvaccinated and partially vaccinated study participants were paradoxically lower compared to those who were fully vaccinated, regardless of type of vaccine. This may be explained by the epidemiologic context in relation to the timing of vaccination, as shown in Fig. 2. There were two COVID-19 surges in the Philippines during the study period—the Delta variant surge in August-October 2021 and the Omicron variant surge in January-February 2022. Of the 64 cases of probable reinfection, 6 (9.4%) occurred during the Delta variant surge while 39 (60.9%) occurred during the Omicron variant surge. During the time of these surges, majority of study participants were already fully vaccinated. Thus, the lower reinfection rates of the unvaccinated and partially vaccinated study participants may reflect the lower incidence of COVID-19 infection in the Philippines during the start of the study period.

Our findings are consistent with other studies that found that higher antibody levels were associated with a lower risk of COVID-19 infection [12]. In our study, those who received mRNA vaccines primary series had the largest rise in antibody titers and correspondingly, the lowest reinfection rate. These findings are also consistent with the results of systematic reviews showing that vaccine effectiveness against COVID-19 infection was highest for the primary series of mRNA vaccine [4, 5].

The mRNA vaccines consist of a lipid nanoparticle enveloping an mRNA molecule that encodes the viral Spike protein. This vaccine induces antigen-specific follicular helper T cell development in the germinal centers of the draining lymph nodes, which would lead to B cell activation, antibody isotype switching, affinity maturation, and formation of plasma cells and memory B cells [13]. This mechanism of action closely resembles the immune response to a natural infection, which may explain why mRNA vaccines stimulate higher antibody titers and consequently, produce greater effectiveness against COVID-19 infection, hospitalization, and death [14].

We also observed that the GMT ratio of all types of vaccine exceeded 4. A four-fold increase in antibody titers is generally the minimum rise interpreted as an “adequate” antibody response [15]. This supports the findings of studies in other countries that the various types of vaccines demonstrate acceptable immunogenicity despite variation in the actual magnitude of humoral response [16]. Among the seven brands of COVID-19 vaccines received by the study participants, only the inactivated Vero cells vaccine had a GMT ratio less than 4. However, only 1 participant received this vaccine.

Among the study participants who received booster doses, the largest GMT ratios were observed among those with inactivated virus vaccine as the primary series, likely due to the lower pre-booster titer compared to those who received viral vectors and mRNA vaccines as primary series. An inverse relationship with pre-immunization titer level and degree of humoral response has been demonstrated in other studies, where a higher pre-vaccination titer is associated with a lower rise in antibody post-vaccination [17].

The GMT ratio was higher with heterologous boosters after inactivated virus and viral vectors primary series compared to homologous boosters. However, among those who received mRNA vaccine as primary series, the GMT ratio was higher for those given homologous boosters compared to heterologous boosters. These findings are consistent with studies in other countries reporting better immunogenicity for heterologous compared to homologous boosters for inactivated virus vaccines, and conversely, better immunogenicity for homologous boosters for mRNA vaccines [18, 19]. The lower GMT ratio of heterologous booster for mRNA vaccine may be due to the use of viral vectors as the booster in 5 out of the 20 participants. As shown in our study and in other published studies, viral vector vaccines generally result in a smaller rise in antibody titers compared to mRNA vaccines. Our findings suggest that the administration of mRNA vaccines as booster, whether as a heterologous booster or homologous booster, results in larger rise in antibody titers.

In this study, adverse events following immunization were more frequently reported among mRNA and viral vector vaccines compared to inactivated virus vaccines. This finding is consistent with other studies [6, 20]. Increased vaccine reactogenicity has been associated with higher post-vaccination antibody levels [21]. This was observed in this study, with participants who received inactivated virus vaccines having the lowest GMT ratio and also the lowest percentage of adverse events following immunization.

Our study had the following limitations. First, in the primary cohort study we conducted, we could not do laboratory confirmation of reinfection due to the unavailability of routine genomic testing for symptomatic patients. Instead, an adjudication committee determined whether reported events were probable reinfections. Hence, the reinfection rates we report in this study refer to probable reinfection rather than confirmed reinfection. Furthermore, reinfection rates in the main cohort study were probably underestimated because testing via RT-PCR or antigen test was encouraged but not provided for free for participants with symptoms consistent with COVID-19. Some symptomatic study participants refused to undergo testing. The study was also unable to detect cases of asymptomatic reinfection. Thus, the reinfection rates reported in this study are likely to be underestimated.

Another limitation is that the antibody titers measured were binding antibodies, not neutralizing antibodies. Tests for neutralizing antibodies are ideal since these are the antibodies that directly interfere the binding and uptake of virus to the host cells [21]. At the time the cohort study was being conducted, there were no certified biosafety level 3 laboratories in the country. However, studies have demonstrated neutralizing antibodies strongly correlate with RBD-specific binding antibodies, and that RBD-specific binding antibody titers can serve as surrogate measures for neutralizing titers [22].

Another limitation in this study is the variation in the timing of antibody titer determination in relation to vaccination, since antibody tests were performed at fixed time points based on the initial COVID-19 infection. Means and standard deviations of the number of interval days between the antibody determination and vaccination were reported to provide appropriate context to the results.

Moreover, the semi-quantitative laboratory test used in the study had an upper limit of detection of 250 U/mL. We performed 10-fold dilution according to manufacturer recommendations to increase the upper limit of detection to 2,500 U/mL. However, several results still exceeded 2,500 U/mL. We performed 100-fold and 1,000-fold dilutions to increase the upper limit of detection to 250,000 U/mL; however, the resulting values at this higher range may have diminished accuracy.

Another limitation is the presence of several confounding variables that affect reinfection rate and antibody titers aside from vaccination. Due to these issues, and the small sample size of the completed cohort study, this study was designed as a descriptive study and the results are intended to be exploratory in nature. Inferential statistics was not done.

Furthermore, the completed cohort study primarily aimed to determine symptoms of COVID-19 reinfection during the follow-up calls. Participants were also asked if they received the COVID-19 vaccine, and the type, brand and date of vaccination. From this recorded data, adverse events following immunization were extracted. However, this is prone to reporting bias. Although COVID-19 symptoms have several similarities as systemic adverse events following immunization, other symptoms such as rashes, flushing or local erythema which were not directly asked by the researchers may have been missed if the information was not volunteered by the study participants. Moreover, data for this study was heavily reliant on the completeness and accuracy of the data recorded in the completed cohort study.

## Conclusion

In a cohort of Filipino individuals previously infected with COVID-19, vaccination with different types and brands of COVID-19 vaccines resulted in varying reinfection rates and increase in antibody titers. We observed the lowest reinfection rate and the highest rise in antibody titers among participants who received the mRNA vaccines as primary series. We observed the highest reinfection rate, the smallest rise in antibody titers, and the lowest percentage of adverse events among participants who received inactivated virus vaccines as primary series. The external generalizability of the results is limited due to several limitations in the main cohort study, including the small sample size and imbalanced distribution in the type of vaccines received.

### Electronic supplementary material

Below is the link to the electronic supplementary material.

Supplementary Material 1

## Data Availability

The datasets used and/or analysed during the current study are available from the corresponding author on reasonable request.

## List of abbreviations

aRR: Adjusted relative risk
CI: Confidence interval
DOH: Department of Health
ECLIA: Electrochemiluminescence immunoassay
GMT: Geometric mean titer
GSD: Geometric standard deviation
HIV: Human immunodeficiency virus
IQR: Interquartile range
RBD: Receptor-binding domain
RR: Relative risk
RT-PCR: Reverse transcription-polymerase chain reaction
SD: Standard deviation
VE: Vaccine effectiveness
