# Supplementary material for: Reinfection rates, change in antibody titers and adverse events after COVID-19 vaccination among patients previously infected with COVID-19 in Metro Manila, Philippines: a secondary analysis of a completed cohort study
Source: BMC Infect Dis. 2023 Nov 1;23:750. doi: 10.1186/s12879-023-08743-6 (PMC10621145; doi:10.1186/s12879-023-08743-6)
Supplement: Supplementary file 1 — Supplementary Material 1 [file 12879_2023_8743_MOESM1_ESM.docx]

APPENDIX 1. Methodology of the completed cohort study

**METHODS**

**Study Design**

We conducted a cohort study to monitor the antibody levels of patients diagnosed with COVID-19. We followed up these patients to determine if there was reinfection within the first year after initial infection.

**Study Setting**

We identified potential study participants from various COVID-19 hospitals and quarantine facilities in Metro Manila. We also invited potential participants to participate in the study by means of posters disseminated in social media platforms. We conducted the study remotely from the University of the Philippines, Manila during the 1-year long follow-up.

We tapped third-party service providers to extract blood specimens from the study participants' homes. Due to the hesitation of certain study participants to healthcare personnel visiting their homes and causing possible negative stigma, other possible venues for blood specimen extractions included the barangay health centers, work address of the study participant, or other areas identified by the study participant. If preferred by the study participant, he/she was brought to the nearest study laboratory (Hi Precision) branch for the scheduled blood extraction. The research team facilitated the arrangement of transportation and shouldered the transportation expense.

**Study Population**

## *Inclusion criteria*

Patients who met the following eligibility criteria were enrolled to the study:

1. Adult (≥18 years old)
2. Diagnosed with COVID-19 through RT-PCR, including patients with asymptomatic, mild, moderate, severe or critical disease
3. Within 21 days since onset of symptoms (if symptomatic) or since RT-PCR positivity (if asymptomatic)
4. Owned a mobile phone
5. Permanent address within Metro Manila
6. Able to provide informed consent

## *Exclusion criteria*

Due to anticipated changes in the circulating antibody levels, participants who received or intended to receive convalescent plasma or intravenous immunoglobulin during the follow-up and monitoring period were excluded from participating.

Participants who received COVID-19 vaccine prior to enrollment were excluded from the study. However, due to ethical reasons, we did not exclude participants who received the SARS-CoV-2 vaccines during the 1-year study follow-up period. Study participants who subsequently received the SARS-CoV-2 vaccine were still included in the study follow-up and determination of antibody levels.

**Study Procedures**

Participants of the current study were followed up for one year, counting from the first day that they showed symptoms of COVID-19 or the day of RT-PCR positivity for asymptomatic patients.

### *Remote Coordination of Study Activities during the COVID-19 Pandemic*

To minimize the risk of infection transmission, the study minimized direct and indirect physical contact between the researchers and study participants. The general approach would be to limit face-to-face interactions with study participants to healthcare workers who are already charged with their clinical care and who are already equipped with the appropriate personal protective equipment (PPE).

The study researchers underwent training on Good Clinical Practice, study-specific consent process and documenting consent, and study-specific conduct of interviews of study participants prior to the start of study implementation. Study researchers operated from a virtual study hub, interacting with study participants through phone calls. Third-party service providers were tapped to facilitate collection of specimens from the participants at the isolation center, at their respective residences or work place, or at barangay health centers. Study participants were brought to the nearest Hi Precision branch if preferred by the study participant. The collected specimens were transported to the study laboratory (main laboratory of Hi Precision). Hi Precision Diagnostics is an accredited medical laboratory that ensures quality control of serum processing for these types of tests.

Healthcare workers were involved in the following study activities:

- Referring potentially eligibility participants to the study staff
- Collecting blood specimens

Study researchers operated from a virtual study hub, interacting with study participants and, in instances where the participant is admitted, the healthcare workers charged with their care, through phone calls. The researchers carried out the following study activities:

- Eligibility screening
- Informed consent process
- Study data collection (at enrollment and follow-up)
- Scheduling and coordination of study-related diagnostic tests
- Tracking of patient location and following-up results of clinician-requested diagnostic test throughout the study

At the start of the study period, COVID-19 cases in Metro Manila were reaching 2,300 to 3,600 cases per day with variants P.1, P.3, B.1.1.7 and B.1.351 detected in the country. We anticipated that 20% of study participants would develop severe disease and require hospitalization. For this subset of patients, the study staff contacted the healthcare worker in the hospital to coordinate scheduled blood extractions and inquire on the results of diagnostic tests that were done as part of the study participant’s clinical care.

For the patients who were discharged from the COVID-19 centers before Day 21, the study staff contacted the study participant or his/her relative to coordinate blood extractions and inquire on the results of diagnostic tests that were done as part of routine clinical care.

The procedures outlined in this section were reviewed and refined periodically, to adapt to the evolving situation in the quarantine facilities and in Metro Manila.

### *Scheduled measurement of SARS-CoV-2 antibodies*

We periodically measured the level of SARS-CoV-2 antibodies at Days 21, (+/- 2 days), 90 (+/- 15 days), 180 (+/- 15 days), 270 (+/- 15 days) and 360 (+/- 15 days) from onset of symptoms or date of RT-PCR positive test for asymptomatic patients. This study used a laboratory-based semi-quantitative test, ECLIA (Elecsys® Anti-SARS-CoV-2 S assay) to measure antibody levels. It detects the RBD-specific total antibody levels (IgG, IgA, IgM). The lower limit of detection of the laboratory test used is 0.4 U/mL, while the upper limit of detection is 250 U/mL. For study participants who had results <0.4 U/mL, the result was recorded as 0.39 U/mL in the database in order to facilitate mathematical computation and data analysis. For study participants with results >250 U/mL, 10-fold dilution was performed to increase the upper limit of detection to 2,500 U/mL.[14] Further dilution was performed as necessary to increase the upper limit of detection to 25,000 and 250,000 U/mL.

Measurement of neutralizing antibodies against SARS-CoV-2 utilizing the plaque reduction neutralization test (PRNT) was originally planned since this is the reference standard. The PRNT quantifies the neutralizing antibody activity against SARS-CoV-2. The test, which measures the level of neutralizing antibodies, is tedious and takes 4 to 5 days to complete. The procedure typically requires the use of live virus, using a specialized set-up in a biosafety level 3 (BSL3) laboratory.[15,16] There are currently no certified BSL3 laboratories in the country.

### *Monitoring for COVID-19 reinfection*

During the one-year follow-up period, we monitored study participants every two weeks to inquire if they developed symptoms consistent with COVID-19, and what the result of the RT-PCR test or SARS-CoV-2 antigen test was, if testing was done.

An adjudication committee held online meetings to classify participants who developed any COVID-19-like symptoms as definite, probable, possible or unlikely to have COVID-19 reinfection. Data presented to the committee members were: demographic information, relevant medical history, date of RT-PCR test indicating COVID-19 infection prior to enrollment, antibody levels before and after symptoms occurred, symptoms, duration of symptoms, history of exposure, type of occupation, RT-PCR test results and cycle threshold values (if available) and vaccination status. The committee members were blinded to the identity of the patient.

There were five members in the adjudication committee, all of whom are clinical epidemiologists. Three of these members were infectious disease specialists, and one was an immunology/allergy specialist. Members could discuss the cases prior to open voting, with the majority vote followed.

## Study Variables

The study investigated the following variables:

At baseline: COVID-19 disease severity, age, co-morbidities

On follow-up**:** SARS-CoV-2 antibody levels, incident COVID-19 (based on self-report of symptoms and laboratory results such as SARS-CoV-2 RT-PCR or antigen test, if available), vaccination status

**Biologic Specimens**

*Blood Samples*

At each blood extraction, 10 ml of whole blood was drawn and placed in non-citrated vials for serum separation. One 5-ml vial each was collected for the following 1) laboratory-based antibody test, and 2) biobanking.

Serum samples were stored and aliquoted in cryotubes at the University of the Philippines National Institutes of Health (UP-NIH) to avoid repeated freeze - thaw cycles. Samples were stored at -70 to -80 degrees Celsius.

*Stored blood samples*

Diagnostic tests to measure SARS-CoV-2 nucleic acids and antibodies continue to evolve. It is anticipated that more advanced laboratory-based diagnostics will become available in the coming months. We obtained written informed consent from the study participants for the storage of their blood samples for future testing..

Blood specimens were stored for future testing, particularly PRNT once it is available. The blood samples collected in this study will be stored at the UP-NIH for a maximum of 25 years, according to the institution’s COVID-19 Samples Storage and Biobanking Policy.

*Specimen collection and transport*

Third-party service providers performed home service collection of blood samples. The transport of specimens from Hi-Precision Diagnostic Center to UP-NIH was done following a Material Transfer Agreement and in accordance with the NIH Protocol for SARS-CoV-2 Specimen Transport.

**Data Collection and Management**

We used a secure data management software (Epidata) for study data collection. User access was restricted through user profiles designated according to user roles. Access to the system was given through individual accounts with password protection. A code assigned to each participant was used in the electronic questionnaires, which is only known to the researcher and the study staff. Electronic data was collated centrally and backed-up every day, at the end of the work day.

Data quality control was implemented by using both preventive and corrective actions. The electronic database, which captured the data electronically, was programmed with data quality rules that automatically perform calculations (e.g. age from birthdate), restrict allowable values to a specific range (e.g. a normal range of values for quantitative laboratory tests), use branching logic (e.g. *If yes* questions), and have mandatory items (i.e. empty response not allowed). At the end of the study, and before performing data analysis, frequency distribution of all variables was examined for out of range values and outliers. Data was also counterchecked from other data sources (e.g. medical records), as applicable. Furthermore, the electronic case forms of a random 10% of all the respondents underwent internal audit by an independent staff member who did not perform data collection to check the accuracy and completeness of the data.

**Data Analysis**

Study data was processed using MS Excel and analyzed using STATA 17 software. Demographic, laboratory, and clinical data were presented using descriptive statistics. Mean with standard deviation (SD), or median and interquartile range (IQR) as appropriate, was used to describe quantitative data. For qualitative data, frequencies were used. Antibody levels were reported as geometric mean titers (GMT) with geometric standard deviation (GSD) at each period of observation, as these are the recommended measures of location and dispersion for antibody titers.[17] Antibody GMTs with GSD were also reported according to initial COVID-19 severity classification and the vaccination status of the participants.

Friedman test was used to compare GMTs across the 5 timepoints. If significant differences was found, pairwise sign test was done at 5% level of significance with adjustments using Bonferroni method ($\alpha=0.05$ divided by 10 pairwise comparisons). The adjusted alpha used was 0.005 and all p-values were compared with the adjusted alpha of 0.005. Kruskal Wallis test was used to compare the GMTs based on severity classification. If significant difference was found, Dunn’s test was done at Bonferonni adjusted level of significance of 0.005 for severity classification ($\alpha=0.05$ divided by all 10 pairwise comparisons).

The incidence of reinfection was estimated at 95% confidence level. Unadjusted and adjusted hazard ratios for the effect of antibody levels on the development of probable reinfection were estimated using Cox proportional hazards model. Antibody level was treated as a continuous variable. The antibody level prior to the reinfection was used for those with probable reinfection. For those without probable reinfection, their GMT across the 5 timepoints were used. Hazard ratios were adjusted for possible confounders including age, sex, co-morbidities, and vaccination status.

**Sample Size Computation**

Liu et al [18] reported a standard deviation of 246 IgG RU/ml for patients with COVID-19 infection on day 14. Using this standard deviation, 244 participants are needed to estimate the mean IgG titer at 99% level and 80% probability of achieving a target width of 88 RU/ml. The level of confidence was adjusted for multiple comparisons by the Bonferonni method since the mean titer will be estimated at 5 periods of observations (alpha=0.05/5=0.01).

Taking into consideration a possible dropout rate of 20%, this study targeted to recruit a total of 307 participants. Dropout is defined as a situation where all outcome data of the participant are missing after a certain timepoint. This includes mortality, withdrawal of consent, and loss to follow-up.

**ETHICAL CONSIDERATIONS**

This study was conducted following the principles outlined in the Declaration of Helsinki, the WHO International Ethical Guidelines for Health-related Research Involving Humans, and the Philippines’ National Ethical Guidelines for Health and Health-Related Research. This research was reviewed and approved by the UP Manila Research Ethics Board. The study protocol was submitted to the UP Manila Institutional Biosafety and Biosecurity Committee for review and clearance. Ethics Review Board (ERB) approval was secured before the start of the study (UPMREB 2020-698-01).

.

*Social Value*

The information from this research has both clinical and public health implications. It is instrumental in providing information regarding immunity to COVID-19, ensure effective public health interventions, and maximize the efficient use of resources, especially once vaccines are available.

*Informed Consent*

Trained study researchers secured Informed consent from each before enrollment into the study. Study researchers underwent training (Good Clinical Practice, study-specific consent process and procedures for documenting consent) prior to the start of study recruitment. Details of the study were discussed and informed consent was secured without deception, coercion, undue influence, or inducement.

We obtained informed consent to participate in the study as soon as a participant becomes eligible to participate, i.e. when an RT-PCR test becomes positive and he/she fulfilled the rest of the inclusion criteria.

The study researchers carried out the informed consent process through a mobile call. This was approved by the Ethics Review Board due to the COVID-19 pandemic. We conducted pre-study training of all researchers in getting the informed consent in order to ensure the quality and standardization of the procedure. Prior to the call, we provided a copy of the study consent form to the potential study participant. Consistent with a face-to-face consent process, we explained the study aims, procedures, and attendant risks and benefits to the participant. We discussed the need for storage of specimens for biobanking of serum for additional quantitative antibody tests (plaque reduction neutralization test, PRNT) with the participant. We secured consent from the study participants for the storage of their blood samples for future testing and documented their consent in the study informed consent form. We encouraged the participant to ask questions on any point that required clarification. We secured participant consent verbally. This was followed by a text message summarizing the main study details and an acknowledgment statement, sent from the study mobile number to the potential participant’s mobile number. Potential participants who consented to participate in the study were asked to send a text reply to the study mobile number. We archived this text reply electronically. We also obtained written informed consent from the study participants.

The total duration of study participation was 52 weeks. Collection of blood samples was conducted on days 21, 90, 180 270, and 360 of study participation (counted starting first day of symptoms or if asymptomatic, from day of RT-PCR positivity).

We informed participants that their participation in the study was voluntary and that they have the right to withdraw at any time of the study. The forms indicated the extent and duration of participation that the study requires.

*Withdrawal Criteria*

As stated in the informed consent form, participants may withdraw their consent to participate in the study at any time. A participant was withdrawn from the study immediately after he/she formalizes the withdrawal of his/her consent to participate.

*Vulnerability*

Patients eligible to join the study may belong to a vulnerable group (e.g. the poor, those lacking social support, the unemployed, and the illiterate). The study staff were trained to explain the study methods, and the risk and benefits of participation in the study in a sensitive and non-threatening way. Care was taken to ensure that consent is given voluntarily, without undue inducement from the study staff.

*Risks, Benefits, and Safety*

This study involved minimal risk. The nature of the risk of participating in the study was similar to the risks typically involved when ongoing routine laboratory check-up or conducting diagnostic tests for an illness, such as the risks of hematoma and pain during venipuncture. However, due to the increased frequency of blood extractions required of participants in the study, the participant’s risk of developing these complications was slightly increased as compared to the general population. We undertook to minimize this risk by tapping on healthcare workers who are professionals in collecting blood samples as part of their routine scope of work. These healthcare workers would have already undergone training on these procedures which was further reinforced through study training before the initiation of the study. The total amount of blood collected at each scheduled extraction was 10 mL for a total of 2 vacutainers. Beyond this, the study did not provide compensation, insurance, or additional healthcare entitlements/treatments. Study participants were informed of the limited measures that the study can provide to address any study-injuries as part of the informed consent process.

Study participants needed to allot time for the scheduled diagnostic tests and to answer the calls of the researchers.

One of the possible benefits of joining the study was the opportunity to gain knowledge of their antibody levels at several time points throughout the year. The results of their respective antibody tests conducted as part of the study was shared with study participants. Should the study participant require hospital admission, the study team also provided assistance in facilitating referral to closely allied healthcare institutions e.g. Philippine General Hospital.

By participating in the study, participants inevitably had to allot time to complete the diagnostic tests at the scheduled time. An allowance of Php 500.00 for each study visit on days 21, 90, 180 270, and 360 requiring blood extraction was provided to them for their time and effort. A cellphone load of Php 50.00 for each scheduled phone call was provided to the participants amounting to a total of Php 1,200 in cellphone load.

*Privacy and Confidentiality*

Confidentiality and anonymity were strictly ensured throughout the conduct of the study in compliance with the Data Privacy Act of 2012. The accomplished questionnaires were kept in a secure, password-protected data management software, compliant with the national regulations on data protection. The names, associated codes, and contact details of the participants were maintained in a master list separate from the study database and available only to the researcher and the study staff. The research data will be stored for a maximum of 20 years.

The names of the participants were not divulged upon reporting results or publication of the research. Participants were also informed through the informed consent form about the measures that were undertaken to maintain their privacy and confidentiality, and that the UP Manila Research Ethics Board or other regulatory authorities might also have access to the information but only for verification of data and procedures.

The electronic documentation of the informed consent was likewise kept in a separate secure, password-protected online file hosting service. The use of mobile applications and computer databases for data collection and storage has implications on the privacy and confidentiality of the participants. Data protection was ensured by the following: (a) participant’s personal information was not encoded in these systems, only their assigned codes, (b) only the principal investigator and the research assistants had access to the systems, and (c) a user management system was put in place to monitor who accessed the system and when do they do it.

Contact between the study personnel and the study participants was limited to phone calls or text messages through SMS or encrypted instant messaging platforms (e.g. Viber or WhatsApp).

*Justice*

The study included all eligible participants fulfilling the inclusion criteria.

*Transparency*

The investigators were transparent about study aspects that may impact the rights, and safety of the participants, or concerning information that may have a bearing on the participants’ informed consent. The study investigators declared any potential conflicts of interest, as summarized in the section below.

*Dissemination and Research Utilization*

The results of this study will be used to improve COVID-19 policies to maximize the efficiency and resources of the national government and add knowledge to COVID-19 evaluation and management in general. Information on the probability of reinfection among previously infected individuals, and whether antibodies can protect against reinfection, is essential in guiding public health policies. The completed report will be published in a peer-reviewed medical journal.
